# A Novel Missense (M206K) STAT3 Mutation in Diffuse Large B Cell Lymphoma Deregulates STAT3 Signaling

**DOI:** 10.1371/journal.pone.0067851

**Published:** 2013-07-04

**Authors:** Guangzhen Hu, Thomas E. Witzig, Mamta Gupta

**Affiliations:** Division of Hematology, Department of Internal Medicine, Mayo Clinic, Rochester, Minnesota, United States of America; University of Nebraska – Lincoln, United States of America

## Abstract

Persistent STAT3 activation has been found in activated B-cell like diffuse large B cell tumors (DLBCL). To investigate whether genetic mutations play a role in aberrant STAT3 signaling in DLBCL, we bi-directionally sequenced all 24 exons of the STAT3 gene in DLBCL tumors (n = 40). We identified 2 novel point mutations in 2 separate (2/40; 5%) patients at exon 7 and 24. Point mutation 2552G>A was a silent mutation in the stop codon. Another heterozygous mutation 857T>A encoded a methionine substitution by lysine at codon 206 (M206K) in the coiled-coil domain of STAT3. We performed site directed mutagenesis to mutate wild type (WT) STAT3α and STAT3β at codon 206 and constructed stable cell lines by lentiviral transfection of STAT3α^WT^, STAT3α^M206K^, STAT3β^WT^ and STAT3β^M206K^ plasmids. The mutation was found to increase STAT3 phosphorylation in STAT3α mutant cell lines with no effect on the STAT3β mutant cell line. Transcriptional activation was also increased in the STAT3α mutant cells compared with STAT3α WT cells as detected by a luciferase reporter assay. Moreover, STAT3α^M206K^ mutant cells were resistant to JAK2 pathway inhibition compared to STAT3α WT cells. These results indicate that missense mutations in STAT3 increase signaling through the JAK/STAT pathway. JAK2 inhibitors may be useful in the patient with this STAT3 mutation as well as those with pathway activation by other mechanisms.

## Introduction

Diffuse large B cell lymphoma (DLBCL) is the most common type of adult non-Hodgkin lymphoma. Aberrant gene expression and mutations are involved in the pathogenesis of DLBCL and may have prognostic and therapeutic relevance. JAK2/STAT3 was shown to be highly activated in DLBCL patient samples and DLBCL cell lines [1], [2], [3]. A high expression of STAT3 protein in DLBCL tumors as detected by immunohistochemistry (IHC) has been associated with unfavorable prognosis in DLBCL in some [4], but not all studies [3]. More importantly, the presence of activated STAT3 in some cases and the availability of inhibitors of the pathway have paved the way for treatment trials [5], [6], [7]. Clarifying the mechanism for STAT3 activation may also assist in choosing patients who may benefit from these targeted therapies. We have demonstrated that elevated serum interleukin 10 (IL-10) is one cause for constitutive STAT3 activation [8]. However, not all DLBCL patients had a high serum IL-10. Mutations specifically missense mutations (which leads to amino acid changes) in JAK2 and STAT3 are another potential cause of STAT3 activation. Our lab has recently showed that there are no JAK2 activating missense mutations in DLBCL tumors [9]. STAT3 mutations have been reported in different kind of hematological malignancies. 30% patients with chronic lymphoproliferative disorders of natural killer cells had STAT3 activating missense mutations [10]. STAT3 mutations have also been described to activate STAT3 in a subset (40%) of patients with T-cell large granular lymphocytic leukemia [11]. The presence of STAT3 mutations in DLBCL tumors has recently described. Morin et al, found that one out of 13 DLBCL tumors had STAT3 mutation [12], similarly Lohr et al found 5 STAT3 mutations from 55 DLBCL cases [13]. However, there is still no data available on the functional relevance of these STAT3 mutations. In this study we detected STAT3 mutations by sequencing in 40 DLBCL patient tumors and determined the functional relevance of STAT3 mutations in DLBCL.

## Materials and Methods

### Patient samples

Cells from 40 DLBCL tumors were obtained from the Iowa/Mayo Lymphoma SPORE Biobank. The Mayo Institutional Review Board (IRB) committee approved the use of human tissue samples for this study. Patients providing written informed consent were eligible for this study if they had a biopsy that upon pathologic review showed DLBCL.

### Cell lines

The OCI-Ly3 (Ly3) and SUDHL2 (DHL2) DLBCL cell lines were a kind gift from Dr. Louis Staudt (NCI, Bethesda) [1]. Ly3 was cultured in Iscove's Modified Dulbecco's Media supplemented with 20% human serum. HEK-293T cell line was from Open Biosystem (Huntsville, AL, USA) and was grown in the Dulbecco's Modified Eagle Medium supplemented with 10% Fetal Bovine Serum.

### Antibodies and reagents

Antibodies of pSTAT3^Y705^, STAT3, HDAC2, GAPDH were from Cell Signaling Technology (Beverly, MA, USA). Beta-Actin antibody was purchased from Santa Cruz (Santa Cruz, CA, USA). Recombinant human IL-10 was from R&D Systems (Minneapolis, MN, USA). TG101348 (TG) was a gift from TargeGEN Pharmaceuticals (now Sanofi-Aventis) (San Diego, CA, USA). TG has now been renamed SAR302503.

### Analysis of STAT3 mutations in DLBCL tumors

We PCR-amplified all 24 exons of the STAT3 gene from the 40 DLBCL tumors. The PCR fragments were sequenced and analyzed at the Mayo Clinic Cancer Center Gene Analysis Core Facility.

### Site directed mutagenesis to create STAT3α and STAT3β mutants

The coding region of STAT3α (NM_139276.2) and STAT3β (NM_213662.1) were amplified and cloned into vector TOPO TA (Invitrogen, Grand Island, NY, US). The 617T>A mutation in the coding region of STAT3α and STAT3β was created with QuikChange II Site-Directed Mutagenesis Kits (Agilent, Santa Clara, CA, USA). WT and mutant genes were then cloned into lentiviral vector pLEX-MCS (Open Biosystem, Huntsville, AL, USA).

### Lentiviral stable transfection

Lentivirus carrying STAT3α^WT^, STAT3α^M206K^, STAT3β^WT^ and STAT3β^M206K^ were made with Trans-Lentiviral ORF Packaging Kit (Thermo Fisher Scientific, Waltham, MA, USA), and then the viruses were transduced into HEK-293T cells. The cells overexpressed with STAT3α^WT^, STAT3α^M206K^, STAT3β^WT^ and STAT3β^M206K^ were screened with puromycin (Invitrogen, Grand Island, NY, USA) and checked for overexpression of STAT3 by western blot.

### Transient transfection

Ly3 cells were transfected with 5 µg of plasmid (pLEX empty vector, pLEX-STAT3α^WT^, pLEX-STAT3α^M206K^, pLEX-STAT3β^WT^ and pLEX-STAT3β^M206K^) using Amaxa Cell Line Nucleofector Kit (Lonza, Walkersville, MD, USA) according to the manufacturer's instruction.

### Western Blotting

The cells were washed with ice-cold PBS before lysate with RIPA buffer (50 mM Tris (pH 7.4), 150 mM NaCl, 1% NP-40, 0.5% sodium deoxycholate, 0.1% SDS, 1 mM EDTA, 15 mM Sodium molybdate) containing Protease Inhibitor Cocktail (Roche Applied Science, Indianapolis, IN, USA), Phenylmethanesulfonyl fluoride (Sigma, St Louis, MO, USA) and Halt Phosphatase Inhibitor Cocktail (Thermo Scientific, Waltham, MA, USA). Proteins were quantified and run on 4–20% Mini-protein gels (Bio-Rad, Hercules, CA, USA), and immunoblotted with antibodies indicated.

### Dual luciferase assay

Plasmid pGL3-rPap1 was a kind gift from Dr. Tavernier (Ghent University, Ghent, Belgium), which was constructed by insertion of a STAT3 responsible promoter, the promoter of rPap1, into pGL3 basic vector [14], [15]. PLEX empty vector, STAT3α^WT^, and STAT3α^M206K^ were transiently transfected together with pGL4-Renilla and pGL3-rPap1 into Ly3 cells. Luciferase activity was measured with dual luciferase assay kit (Promega, Madison, WI, USA). Firefly luciferase activity of pGL3-rPap1 was normalized by Renilla luciferase activity of pGL4-Renilla, which was used as a transfection efficiency control.

### Nuclear and cytoplasmic analysis

Nuclear and cytoplasmic extracts were separated with NE-PER nuclear and cytoplasmic extraction kit (Thermo Scientific, Waltham, MA, USA) as manufacturer's instruction.

### Proliferation assay

After transfection with pLEX empty vector, pLEX-STAT3α^WT^ and pLEX-STAT3α^M206K^, Ly3 cells (0.1 million/200 µl/well) were seeded into 96-well plate in 4 replicates and proliferation was assessed by ^3^H-thymidine incorporation assay as described before [16].

## Results

### Identification of STAT3 mutations in DLBCL tumors

**Figure 1:**
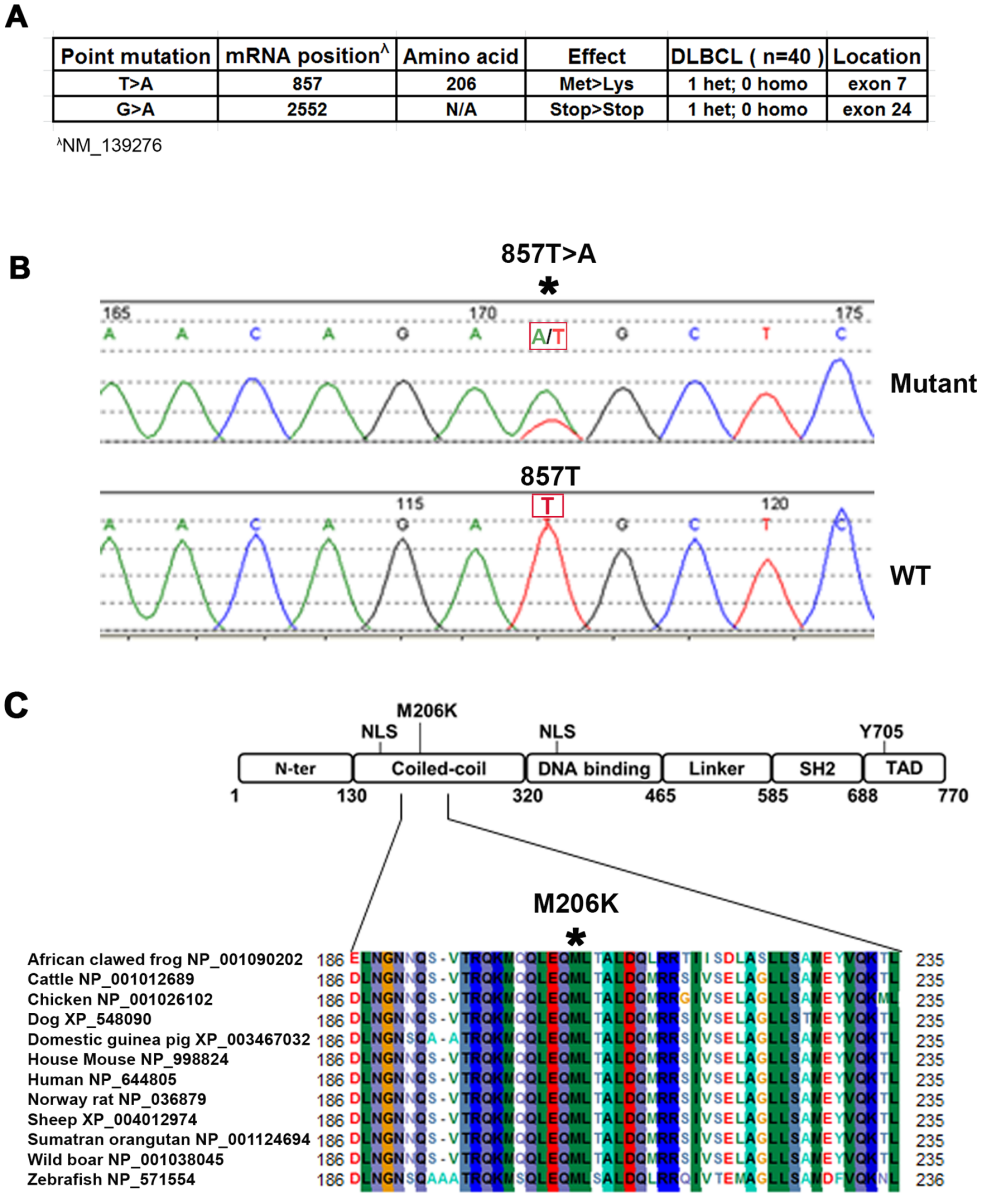
Identification of STAT3 mutations in DLBCL tumors. (**A**) Data summarizing STAT3 mutations in DLBCL tumors (n = 40) (**B**) Chromatograms of STAT3 DNA sequence showing 857T>A heterozygous mutation. (**C**) Modular structure of STAT3 showing that Methionine 206 is located in the coiled-coiled domain of STAT3. Alignment of part of STAT3 protein sequence from various species showing that methionine 206 is a conserved amino acid.

To identify STAT3 mutations in DLBCL, we sequenced all exons of the STAT3 gene from 40 DLBCL tumors. Two novel point mutations, 857T>A and 2552G>A, were identified in two separate tumors. The point mutation 857T>A was located in the exon 7 and predicted a missense substitution of methionine by lysine at codon 206 (M206K) (**Figure 1A**). Chromatograms showed a single peak of nucleotide thymidine (T) at position 857 in WT STAT3 while there were both nucleotide adenosine (A) and T peaks at position 857 in mutant STAT3. These results indicated that the mutation was heterozygous (**Figure 1B** **)**. Modular structure indicates that this M206K STAT3 mutation is located near a nuclear localization signal (NLS) in the coiled-coil domain of STAT3 (**Figure 1C**). The coiled-coil domain of STAT3 is essential for STAT3 nuclear translocation and retention [17], [18], [19], and is also involved in the recruitment of STAT3 to receptors when activated by cytokines [15], [20]. Alignment analysis showed that Met206 (M206) is a conserved amino acid across STAT3 proteins from different species (**Figure 1C**).

**Figure 2:**
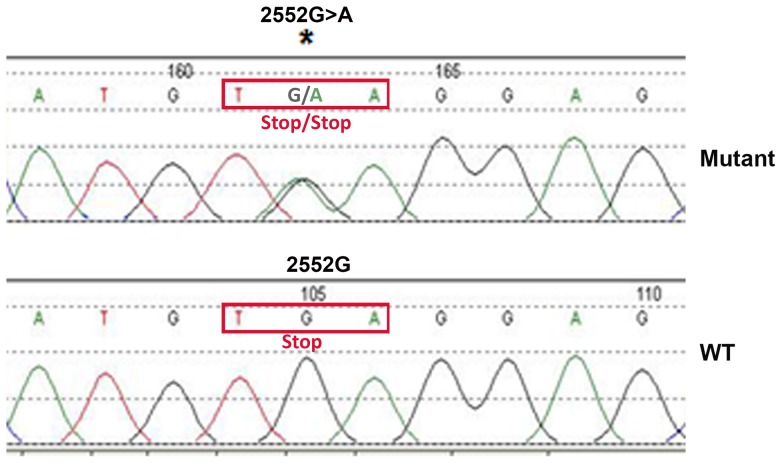
Identification 2552G>A mutation in STAT3 in DLBCL tumors. Chromatograms of part of the patient STAT3 DNA sequence show 2552G>A mutation.

Another STAT3 mutation 2552G>A was located in the stop codon and didn't predict a missense substitution. Chromatogram shows that the nucleotide at position 2552 is guanosine (G) in WT STAT3 and both G and A in mutant STAT3 (**Figure 2**), suggesting that it is a heterozygous mutation. The 2552G>A mutation leads to the stop codon TGA change to TAA, which is also a stop codon (**Figure 2**). This suggests that 2552G>A mutation is a synonymous mutation.

### Effect of STAT3^M206K^ mutation on STAT3 signaling

**Figure 3:**
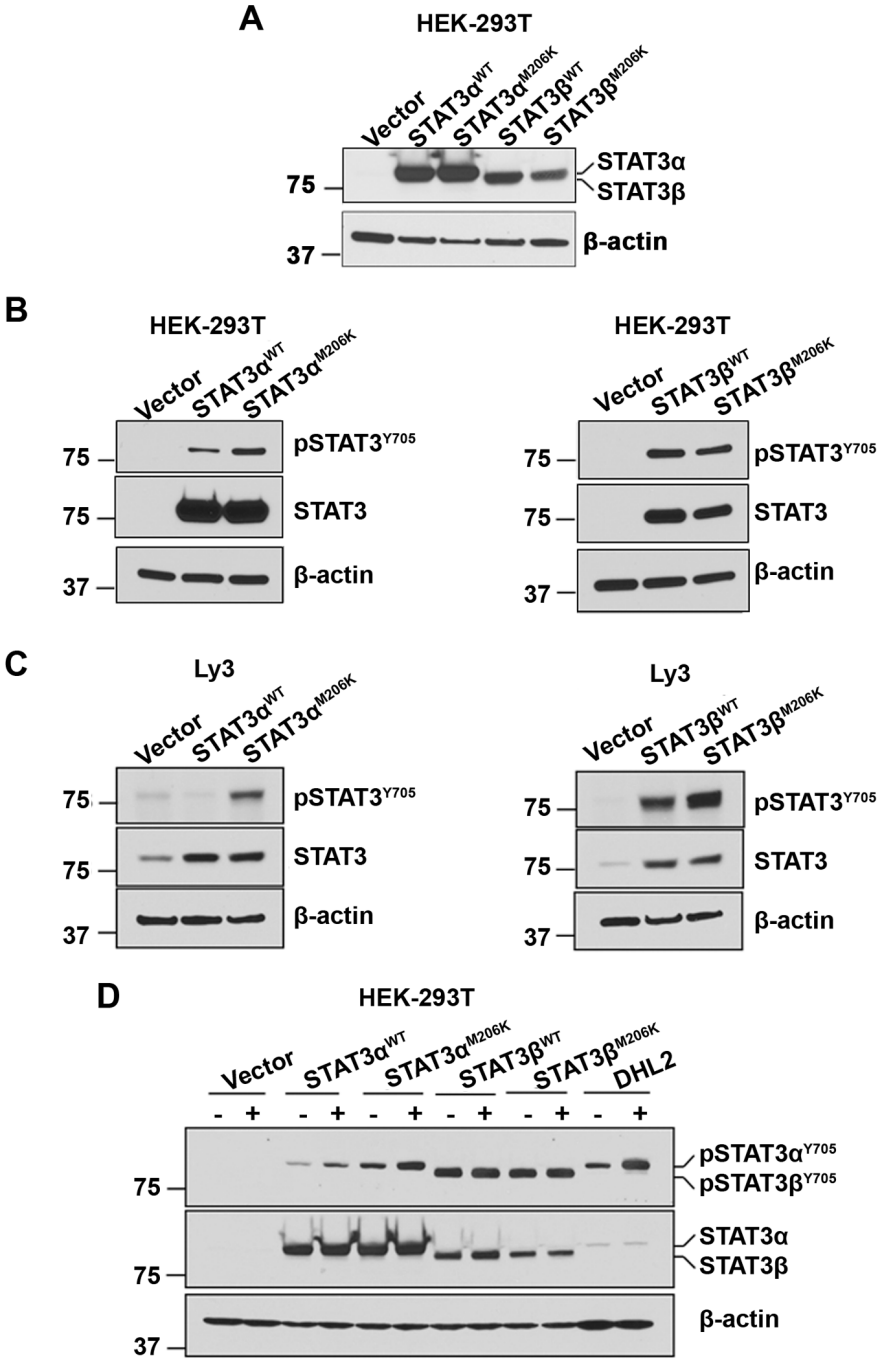
The effect of STAT3 (M206K) mutation on STAT3 signaling. (**A**) Western blot (n = 4) analysis shows overexpression of STAT3 in stably transfected HEK-293T cells by STAT3α^WT^, STAT3β^WT^, STAT3α^M206K^ and STAT3β^M206K^ plasmids. (**B**–**C**) STAT3 phosphorylation was assessed in STAT3α^WT^, STAT3β^WT^, STAT3α^M206K^ and STAT3β^M206K^ stably transfected HEK-293T cells by western blotting (n = 3) (**B**) and transiently transfected Ly3 cells (n = 2) (**C**)**.** (**D**) STAT3 phosphorylation (n = 2) was assessed in response to IL-10 in HEK-293T cells stably transfected with STAT3α^WT^, STAT3β^WT^, STAT3α^M206K^ and STAT3β^M206K^ plasmids. DHL2 cells were used as a positive control for IL-10 treatment.

To confirm whether the STAT3^M206K^ mutation affected the activation of STAT3, lentiviral vectors pLEX-STAT3α^WT^, pLEX-STAT3α^M206K^, pLEX-STAT3β^WT^ and pLEX-STAT3β^M206K^ were constructed and stably overexpressed in HEK-293T cells (**Figure 3A**). Sequencing results showed that the nucleotide was T at position 857 in WT STAT3, while the nucleotide was A at position 857 in mutant STAT3, suggesting that the nucleotide at position 857 was successfully mutated (**Figure S1**).

As phosphorylation at tyrosine 705 is essential for activating STAT3, we checked the effect of STAT3^M206K^ mutation on the STAT3 tyrosine phosphorylation (pSTAT3). **Figure 3B** shows that mutant STAT3α^M206K^ stably transfected HEK-293T cells have a higher level of pSTAT3 as compared to WT STAT3α^WT^ and vector alone-transfected cells. However, the pSTAT3 level was similar in both WT STAT3β^WT^ and mutant STAT3β^M206K^ transfected cells (**Figure 3B**). Next, we transiently transfected pLEX-STAT3α^WT^, pLEX-STAT3α^M206K^, pLEX-STAT3β^WT^ and pLEX-STAT3β^M206K^ plasmids into the Ly3 DLBCL cell line. Western blot analysis showed that the pSTAT3 level was higher in mutant STAT3α^M206K^ transfected cells compared to WT STAT3α^WT^ transfected cells, while the pSTAT3 level was similar in both WT STAT3β^WT^ and mutant STAT3β^M206K^ transfected cells (**Figure 3C**). These data suggest that the STAT3^M206K^ mutation activates STAT3 signaling through STAT3α but not STAT3β. Cytokines can also activate STAT3 and IL-10 plays a role in STAT3 activation in DLBCL cells [8]. To learn whether the STAT3^M206K^ mutation affects the response of STAT3 to cytokine, we treated the stably transfected HEK-293T transfected cells with IL-10 and found that the pSTAT3 level in STAT3α^M206K^ mutant cells was increased with IL-10 treatment as compared with WT STAT3α cells. However, both STAT3β^WT^ and STAT3β^M206K^ transfected cell lines did not respond to IL-10 treatment (**Figure 3D**). These data suggest that the STAT3^M206K^ mutation enhances STAT3α signaling in the presence of IL-10.

### Effect of STAT3^M206K^ mutation on STAT3 transactivation

**Figure 4:**
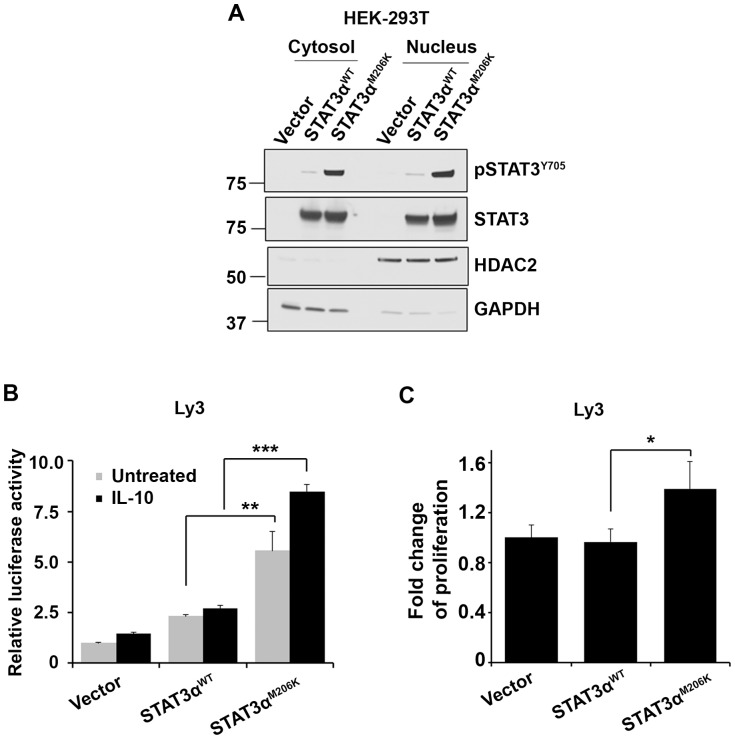
The effect of the STAT3 ^M206K^ mutation on STAT3 translocation to the nucleus, transactivation activity, and cell proliferation. (**A**) STAT3 phosphorylation (n = 3) was assessed in both nucleus and cytosol in stably transfected HEK-293T cells by mutant STAT3α^M206K^ and WT STAT3α plasmids. HDAC2 was used as a marker for nuclear protein, and GAPDH was used as a marker for cytoplasmic protein. (**B**) The transactivation activity of WT STAT3α^WT^ and mutant STAT3α^M206K^ was evaluated by luciferase reporter assay in Ly3 cells. Briefly, Ly3 cells were transiently transfected with plasmids STAT3α^WT^ and STAT3α^M206K^ along with a STAT3 luciferase reporter followed by IL-10 treatment for 6 hours and cells were used for luciferase assay. Bars represent mean ± SD from 3 replicates. Data was repeated three times (**P = 0.0042; ***P<0.001). The data were analyzed by the two-tailed unpaired Student's t test. (**C**) The effect of WT STAT3α^WT^ and mutant STAT3α^M206K^ on Ly3 cells proliferation by H^3^-thymidine incorporation assay. Bars represent mean ± SD from 4 replicates. The experiment was repeated three times (*P = 0.013). The data were analyzed by the two-tailed unpaired Student's t test.

Activated STAT3 needs to translocate to the nucleus in order to activate target gene transcription. To determine if the STAT3^M206K^ mutation can increase the nuclear translocation of activated STAT3, we measured pSTAT3 levels in the nucleus and cytosol and found the pSTAT3 level was higher in both compartments in the STAT3α^M206K^ transfected cells compared to STAT3α^WT^ transfected cells (**Figure 4A**). Next, in order to determine if nuclear STAT3α^M206K^ can increase the transactivation activity of STAT3α, a STAT3-driven luciferase reporter construct along with pLEX-STAT3α^WT^ or pLEX-STAT3α^M206K^ was transfected into Ly3 cells. We found that STAT3 transactivation activity was higher in mutant STAT3α^M206K^ transfected cells in response to IL-10 as compared to that in WT STAT3α^WT^ transfected cells (**Figure 4B**). Constitutively activated STAT3 can promote cell proliferation in DLBCL [2]. By use of H^3^-thymidine incorporation assay we determined that mutant STAT3α^M206K^ further increased the proliferation of Ly3 cells compared to WT STAT3α^WT^ (**Figure 4C**). These data indicate that the STAT3^M206K^ mutation has functional consequences by activating the transactivation activity of STAT3α and increasing cell proliferation.

### Effect of STAT3α^M206K^ mutation on JAK2 inhibition

**Figure 5:**
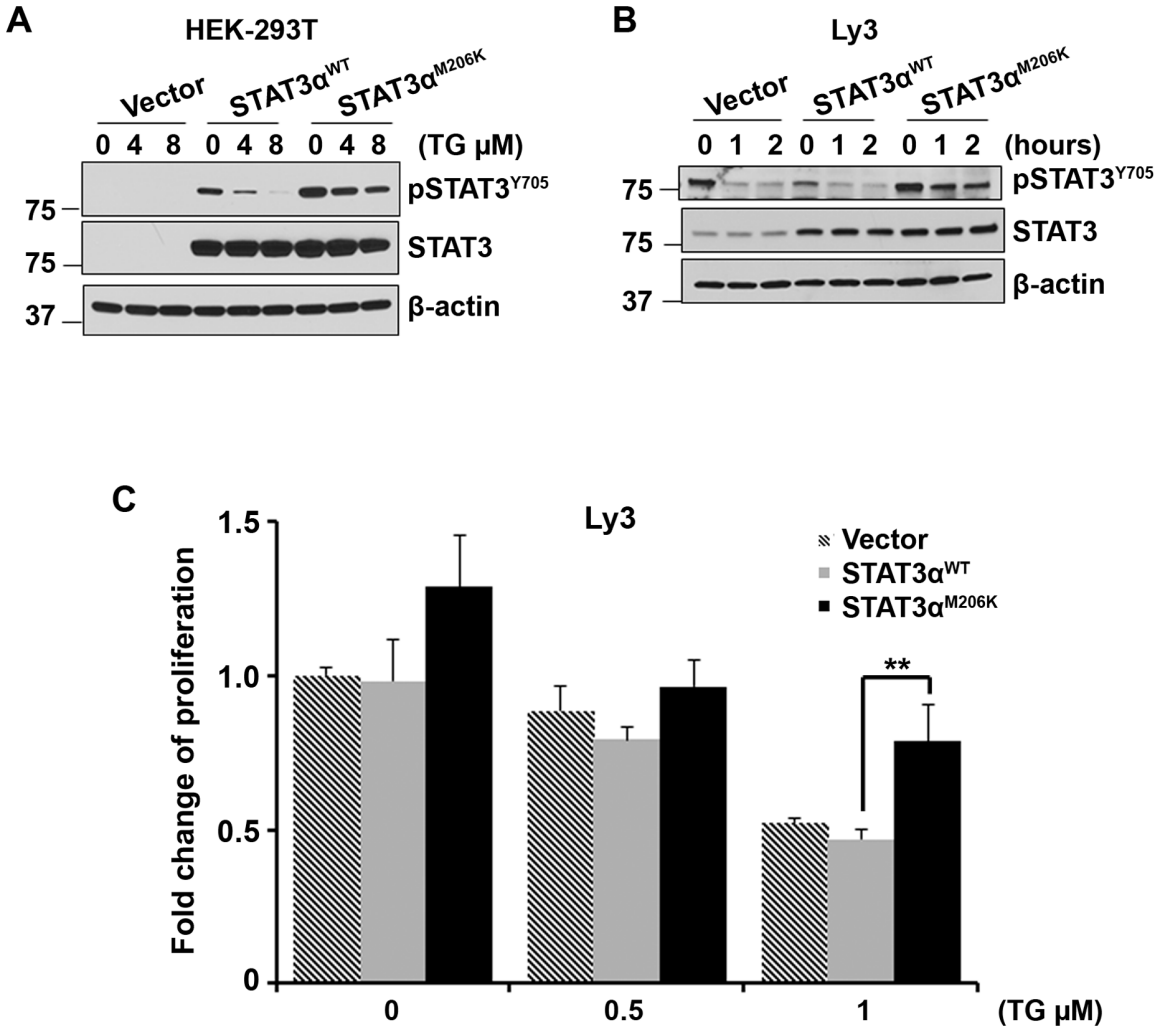
The effect of STAT3α M206K mutation on the sensitivity of DLBCL cells to JAK2 inhibitor TG101348. (**A–B**) The effect of JAK2 inhibitor TG101348 on STAT3 dephosphorylation was assessed in WT STAT3α^WT^ and mutant STAT3α^M206K^ in stably transfected HEK-293T cells (**A**) and transiently transfected Ly3 cells (**B**). Data were repeated three times. (**C**) The effect of the overexpression of WT STAT3α^WT^ and mutant STAT3α^M206K^ on cell proliferation in the presence or absence of JAK2 inhibitor TG101348. Bars represent mean ± SD from 4 replicates. The experiment was repeated two times (**P = 0.0023). The data were analyzed by the two-tailed unpaired Student's t test.

To test whether the deregulated STAT3 signaling by STAT3^ M206K^ mutation can be blocked by an inhibitor of the JAK2 signaling pathway, we treated STAT3α^WT^ and STAT3α^M206K^ stably transfected HEK-283T cells with TG101348, an inhibitor of JAK2 [21], [22], [23]. As expected, WT STAT3α^WT^ cells were very sensitive to JAK2 inhibition and pSTAT3α was completely inhibited with TG101348 treatment. However STAT3α^M206K^ mutant cells were resistant to JAK2 inhibition and TG101348 treatment was not able to completely dephosphorylate STAT3 (**Figure 5A**). More or less similar results were found when we transiently transfected WT STAT3α^WT^ and mutant STAT3α^M206K^ into the Ly3 cells and treated with JAK2 inhibitor (**Figure 5B**). These data suggest that mutant STAT3α^M206K^ are resistant to JAK2 inhibition.

We then checked whether overexpression of the STAT3α^M206K^ mutant could rescue the JAK2 inhibition mediated cell growth. Proliferation of mutant STAT3α^M206K^ transfected Ly3 were less inhibited compared to that of vector only or WT STAT3α^WT^ transfected cells specifically at 1 µM TG101348 concentration (**Figure 5C**). When treated with 1 µM TG101348, approximately 50% of the cell proliferation was inhibited in WT STAT3α^WT^ transfected cells, while only 20% of the cell proliferation was inhibited when mutant STAT3α^M206K^ was transfected into the cells (**Figure 5C**). These data suggest that mutant STAT3α^M206K^ can partially rescue the JAK2 inhibition mediated cell growth.

## Discussion

There are several potential mechanisms for aberrant JAK2/STAT3 activation in DLBCL tumors including cytokines such as IL-10 [8], loss of negative regulators such as tyrosine phosphatases, and genetic mutations. We have previously demonstrated that JAK2 mutations are not the cause [9]; however, there have been few reports on the role of STAT3 mutations in lymphoma in general and specifically DLBCL. Overall, we found two point mutations in STAT3 gene in 40 DLBCL tumors (5%) indicating that mutations in STAT3 in DLBCL tumors are uncommon. Out of these 2 STAT3 mutations only one mutation (M206K) was identified as a missesne mutation that was located in coiled-coil domain of STAT3 protein. Pasqualucci et al, found no somatic STAT3 mutations in their studies using next-generation sequencing on tumor cells from six DLBCL patients [24]. Morin et al found one of 13 cases studied to have a missense mutation that resulted in mutation of serine to arginine at codon 614 [12]. Lohr et al. sequenced 55 DLBCL cases and found 5 that had STAT3 mutations [13]. Four of these were missense mutations (R152W, K658R, H447Y, R278H) and the other case had a deletion, E616del. A previous study reported a STAT3 missense mutation (R382Q) in a DLBCL patient with hyper-immunoglobulin E syndrome (Hyper IgE) [25]. Interestingly, 2 out of the 7 missense mutations (28%) reported so far in DLBCL were located in the coiled-coil domain. Although there have been 6 reported cases of missense STAT3 mutations in patients with DLBCL, there have been no reports of the functional relevance of these mutations. We have demonstrated that the M206K STAT3 mutation in DLBCL was able to activate STAT3 signaling resulting in an increase cell proliferation. The mutation also made the cells more resistant to JAK2 inhibition with the novel catalytic site JAK2 inhibitor TG101348 now in clinical trial for myeloproliferative neoplasms. STAT3 mutations are thus another potential mechanism of STAT3 activation in DLBCL. Although we have shown the functional relevance of the M206K STAT3 mutation, the functional consequences of other STAT3 mutations have not been described to date.

## Supporting Information

Figure S1 **Confirmation of STAT3 857T>A mutation by sequencing of pLEX-STAT3α^M206K^ and pLEX-STAT3β^M206K^ plasmids.** Sequence alignment and chromatograms of part of the sequencing result showing that the nucleotide is T at position 857 in WT STAT3 and A at position 857 in mutated STAT3. NM_139276 is WT STAT3 gene from NCBI.
(TIF)
