# Supplementary figures and images for: A Novel Missense (M206K) STAT3 Mutation in Diffuse Large B Cell Lymphoma Deregulates STAT3 Signaling
Source: PLoS One. 2013 Jul 4;8(7):e67851. doi: 10.1371/journal.pone.0067851 (PMC3701620; doi:10.1371/journal.pone.0067851)

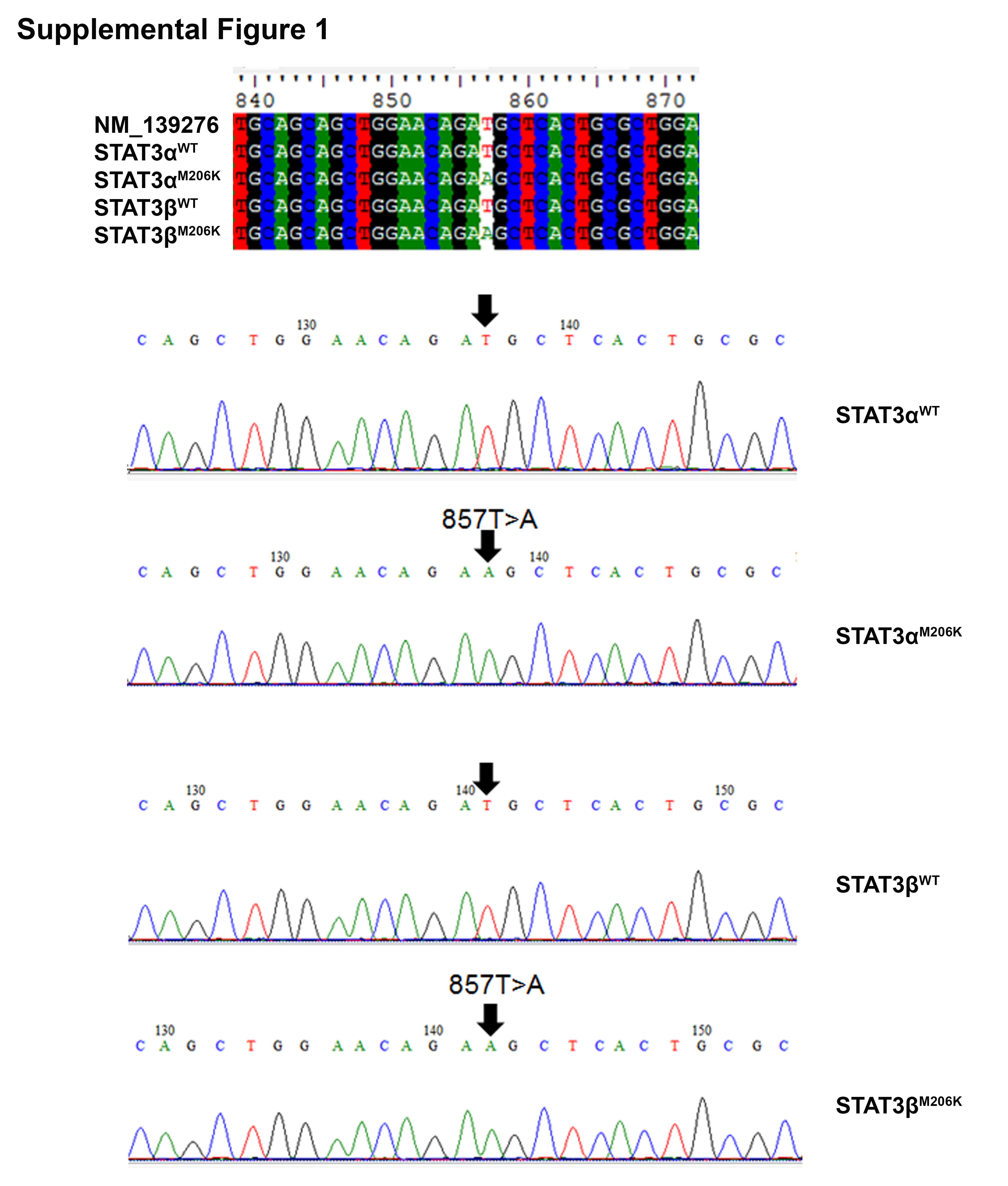

Supplement: Figure S1 — Confirmation of STAT3 857T>A mutation by sequencing of pLEX-STAT3αM206K and pLEX-STAT3βM206K plasmids. Sequence alignment and chromatograms of part of the sequencing result showing that the nucleotide is T at position 857 in WT STAT3 and A at position 857 in mutated STAT3. NM_139276 is WT STAT3 gene from NCBI. (TIF) [file pone.0067851.s001.tif]
